# Nonmuscle myosin IIA is involved in recruitment of apical junction components through activation of α-catenin

**DOI:** 10.1242/bio.031369

**Published:** 2018-04-13

**Authors:** Masayuki Ozawa

**Affiliations:** Department of Biochemistry and Molecular Biology, Graduate School of Medical and Dental Sciences, Kagoshima University, 8-35-1 Sakuragaoka, Kagoshima 890-8544, Japan

**Keywords:** Adhesion, α-Catenin, Cadherin, Cell junctions, Nonmuscle myosin IIA

## Abstract

MDCK dog kidney epithelial cells express two isoforms of nonmuscle myosin heavy chain II, IIA and IIB. Using the CRISPR/Cas9 system, we established cells in which the IIA gene was ablated. These cells were then transfected with a vector that expresses GFP–IIA chimeric molecule under the control of a tetracycline-responsible element. In the absence of Dox (doxycyclin), when GFP–IIA is expressed (GFP–IIA+), the cells exhibit epithelial cell morphology, but in the presence of Dox, when expression of GFP–IIA is repressed (GFP–IIA−), the cells lose epithelial morphology and strong cell–cell adhesion. Consistent with these observations, GFP–IIA− cells failed to assemble junction components such as E-cadherin, desmoplakin, and occludin at cell–cell contact sites. Therefore, IIA is required for assembly of junction complexes. MDCK cells with an ablation of the α-catenin gene also exhibited the same phenotype. However, when in GFP–IIA− cells expressed α-catenin lacking the inhibitory region or E-cadherin/α-catenin chimeras, the cells acquired the ability to establish the junction complex. These experiments reveal that IIA acts as an activator of α-catenin in junction assembly.

## Abstract (precis)

**Summary:** The establishment and regulation of cell adhesion are fundamental to the development and maintenance of tissue organization in multicellular organisms. The role of nonmuscle myosin IIA is studied.

## INTRODUCTION

The establishment and regulation of cell adhesion are fundamental to the development and maintenance of tissue organization in multicellular organisms. In epithelia, cell–cell adhesion is mediated primarily through adherens junctions (AJs) and desmosomes. Because of the importance of cell–cell adhesion in development and tissue function in adults, it is critical to understand the mechanisms by which cells regulate the assembly and disassembly of adhesive junctions.

Cadherins comprise a family of transmembrane cell-surface glycoproteins that mediate Ca2^+^-dependent cell–cell adhesion (Takeichi, 2014). In epithelial cells with well-developed intercellular junctions, E-cadherin is concentrated in the AJs but appears to influence other intercellular junctions such as tight junctions (TJs) and desmosomes (Gumbiner et al., 1988; Watabe et al., 1994; Amagai et al., 1995). Dysfunction of E-cadherin has been implicated in the invasiveness and carcinogenesis of tumor cells and human tumors (Vleminckx et al., 1991; Schipper et al., 1991; Berx et al., 1995).

Cadherins are transmembrane receptors that mediate homophilic intercellular adhesion via their extracellular domains and recruit protein partners to their cytoplasmic domains. Intracellularly, they interact with a group of proteins, collectively termed catenins (Ozawa et al., 1989). α-catenin (Herrenknecht et al., 1991; Nagafuchi et al., 1991) links cadherin/β-catenin or cadherin/plakoglobin complexes to F-actin, either directly (Rimm et al., 1995) or indirectly via α-actinin (Knudsen et al., 1995), vinculin (Watabe-Uchida et al., 1998), formin (Kobielak et al., 2004), or EPLIN (epithelial protein lost in neoplasm) (Abe and Takeichi, 2008). Although biochemical studies had challenged the notion that the cadherin–catenin complex binds directly to F-actin (Drees et al., 2005; Yamada et al., 2005), recent studies revealed strong and stable bonds between the cadherin–catenin complex and an actin filament form under force (Buckley et al., 2014). Thus, monomeric α-catenin bound to the cadherin/β-catenin complex mediates the physical linkage between AJs and the actin cytoskeleton (Desai et al., 2013), and cadherin is constitutively under actomyosin-generated tension (Smutny et al., 2010; Borghi et al., 2012). Moreover, α-catenin has been proposed to act as a mechanosensor in AJ formation by facilitating the recruitment of vinculin to AJs in a manner dependent upon actomyosin-derived tension (Yonemura et al., 2010). Force-dependent conformational changes in α-catenin regulate its binding to vinculin, an actin-binding protein, and reinforce intercellular adhesion (Yonemura et al., 2010; le Duc et al., 2010; Ishiyama et al., 2013; Yao et al., 2014; Kim et al., 2015). Moreover, as demonstrated using a fluorescence resonance energy transfer (FRET) tension sensor, the actomyosin cytoskeleton exerts tensile forces on E-cadherin in an α-catenin-dependent manner (Borghi et al., 2012). Taken together, these observations show that α-catenin is a key mechanosensory protein involved in transmitting actomyosin cytoskeletal tension to the cell membrane.

Nonmuscle myosin II (NMII) is a myosin that constitutes several percent of total cellular protein (Vicente-Manzanares et al., 2009), and NMII isoforms are widely expressed in embryonic and adult tissues (Golomb et al., 2004). They play distinct but related roles in cell biology, including processes such as motility (Maupin et al., 1994; Wylie and Chantler, 2001; Kolega, 2003), adhesion (Conti and Adelstein, 2008; Wang et al., 2010), and vesicle trafficking (Togo and Steinhardt, 2004). Experiments using a highly selective inhibitor, blebbistatin (Straight et al., 2003), or siRNA-mediated knockdown, have shown that NMII activity is required for accumulation and stability of cadherins at junctions (Shewan et al., 2005; Miyake et al., 2006; Lambert et al., 2007). Genetic ablation of NM heavy chain IIA (IIA) from embryonic stem cells and mouse embryos results in a loss of cell–cell adhesion, which correlates with a decrease in E-cadherin and β-catenin localization at cell–cell contacts (Conti et al., 2004). Knockdown of NMIIA by siRNA decreased both the amount of E-cadherin at cell–cell contacts and cadherin-based adhesion (Smutny et al., 2010). Thus, NMIIA activity is needed for E-cadherin localization to the junctions. These studies suggest that tension on actin provided by NMII is necessary for maintaining the proper localization of these cell adhesion proteins. On the other hand, NMII has been reported to be involved in disassembly of AJs, and blebbistatin prevents disassembly of AJs in calcium-depleted cells (Ivanov et al., 2004).

To address the functional role of NMIIA in the assembly of junction complex components, we ablated its heavy chain gene using the CRISPR/Cas9 system, and then introduced an expression vector for GFP-tagged IIA (GFP–IIA) under the control of the tetracycline (Tet) promoter. We analyzed the assembly of the components of AJs, as well as other junctions, in the presence or absence of Dox. In the absence of Dox, when GFP–IIA was expressed (GFP–IIA+ cells), the cells exhibited epithelial cell morphology, but in the presence of Dox, when expression of GFP–IIA was repressed (GFP–IIA− cells), the cells lost this morphology. Consistent with these observations, GFP–IIA− cells failed to assemble junction components such as E-cadherin, desmoplakin, and occludin at cell–cell contact sites. Therefore, IIA is required for assembly of junction complexes. However, when α-catenin constructs with deletions were expressed in GFP–IIA− cells, the cells were rescued, and acquired the ability to establish the junction complex, indicating that these deletions activate α-catenin. These experiments reveal that (1) α-catenin is, indeed, the downstream target of IIA in junction assembly, and (2) IIA acts as an activator of α-catenin in junction assembly.

## RESULTS

### Establishment of MDCK cells lacking the IIA gene and expressing a GFP–IIA chimeric molecule under the control of the tetracycline-responsive element

In this study, we used a MDCK (type II) clone T23 expressing the Tet repressor (Barth et al., 1997). We first established MDCK cells in which the IIA gene was ablated, using the CRISPR/Cas9 system (Jinek et al., 2012; Cong et al., 2013; Mali et al., 2013), and then introduced the GFP-tagged IIA construct (Wei and Andersen, 2000) expressed under the control of the Tet-repressible element (TRE); in the presence of either Tet or doxycycline (Dox), expression of exogenous protein was completely repressed (Gossen and Bujard, 1992).

**Fig. 1.**
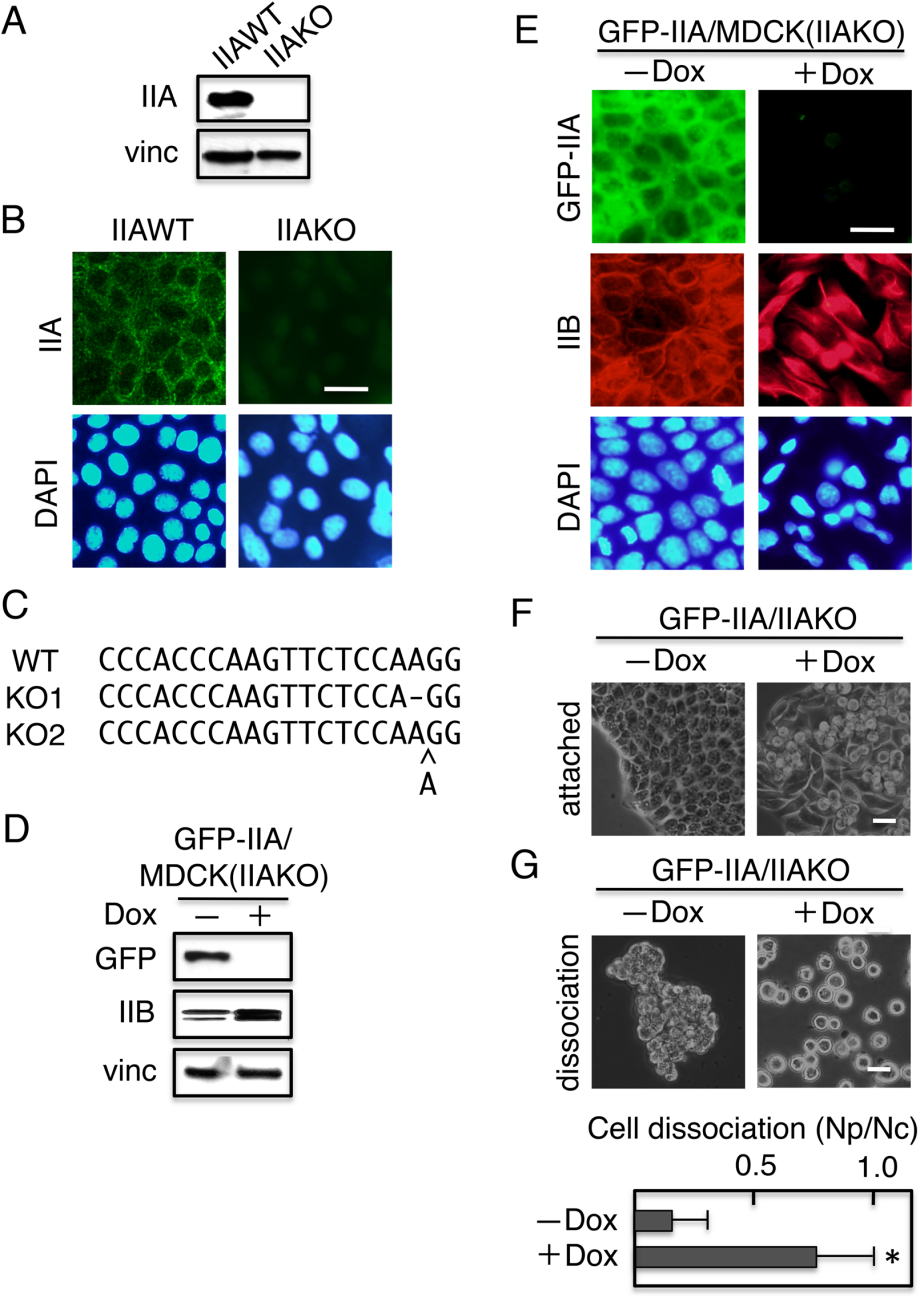
**Establishment of MDCK cells lacking the NMHCIIA gene and expressing a GFP-NMHCIIA chimeric molecule under the control of the tetracycline-responsive element.** Using the CRISPR/Cas9 system, NMHCIIA (IIA) gene of MDCK cells was ablated, yielding IIAKO cells, which were transfected with a vector encoding GFP-tagged NMHCIIA (GFP–IIA) under the control of the TRE, yielding GFP–IIA/IIAKO cells. (A) Immunoblot detection of IIA and vinculin (vinc) in IIAWT and IIAKO cells. Vinculin was used as a loading control. (B) Cells were stained with anti-IIA or DAPI. (C) Genomic sequencing revealed mutations in the target regions of the NMHCIIA gene that induced frame-shift mutations. Of six independent clones sequenced, a 1-bp deletion was detected in two clones, and a 1-bp insertion was identified in four clones. (D,E) Dox-repressible expression of GFP–IIA in GFP–IIA/IIAKO cells. Cells were cultured for 4 days with (+) or without (−) Dox. (D) The cells were subjected to immunoblot analysis with the indicated antibodies. GFP–IIA was detected with an anti-GFP antibody. Vinculin was used as a loading control. (E) GFP–IIA was detected as GFP fluorescence and cells were stained with anti-IIB or DAPI. (F) Cell morphology. Cells cultured with (+) or without (−) Dox were observed using a phase-contrast microscope. (G) Dissociation assays. Cells were cultured in the presence (+) or absence (−) of Dox prior to the assay. (Upper panels) Phase-contrast micrographs. Cells treated with Dox lost the mechanical integrity of their cell sheets, but untreated cells did not. (Bottom panels) Quantification of cell dissociation assays. The extent of cell dissociation is represented by the index Np/Nc, where Np and Nc are the total numbers of particles and cells per dish, respectively. Values represent the mean±s.e.; *n*=at least 3 times. *P<0.01 compared with control (−Dox). Scale bars: 25 µm.

Immunoblotting (Fig. 1A) and immunofluorescence staining (Fig. 1B) with antibodies against IIA confirmed that a selected MDCK clone in which the IIA gene was ablated (IIAKO) lost expression of IIA. Consistent with these results, sequencing of the genomic DNA isolated from the clone revealed either a 1-bp deletion or 1-bp addition, resulting in a frame-shift mutation in the IIA gene (Fig. 1C). The MDCK clone was then transduced with the GFP–IIA construct under the control of the TRE; the resultant clones were termed GFP–IIA/IIAKO. The cells were cultured for 4 days without or with Dox (−/+ Dox), and then analyzed for expression of GFP–IIA (Fig. 1D). GFP–IIA was detected in protein extracts from the cells cultured without Dox, but not in those from the cells cultured with Dox.

### GFP–IIA-dependent localization of junction components to cell–cell contact sites in IIAKO cells

GFP–IIA/IIAKO cells cultured in the absence of Dox expressing GFP–IIA grew in monolayer cultures as epithelial clusters with a typical cobblestone morphology (Fig. 1E). Culturing the cells in the presence of Dox, causing almost complete repression of GFP–IIA expression, induced dramatic changes, including multilayering of cells, fibroblast-like elongation of cells in the basal layer, and rounding of cells in the top layer (Fig. 1E).

Concomitant with these morphological changes, cells cultured in the presence of Dox lost the integrity of the epithelial sheet. Cell sheet integrity was determined using the Dispase-based dissociation assay, which was originally developed to determine the mechanical integrity of epidermal keratinocytes, cells that endure mechanical stress (Ishii et al., 2005; Simpson et al., 2010). Although cells cultured in the absence of Dox were resistant to mechanical dissociation, cells cultured in the presence of Dox were dissociated into single cells or small aggregates of cells after detachment from the dishes by Dispase and subsequent mechanical dissociation by pipetting (Fig. 1F). Thus, loss of GFP–IIA expression disrupted the mechanical integrity of the cell sheets.

**Fig. 2.**
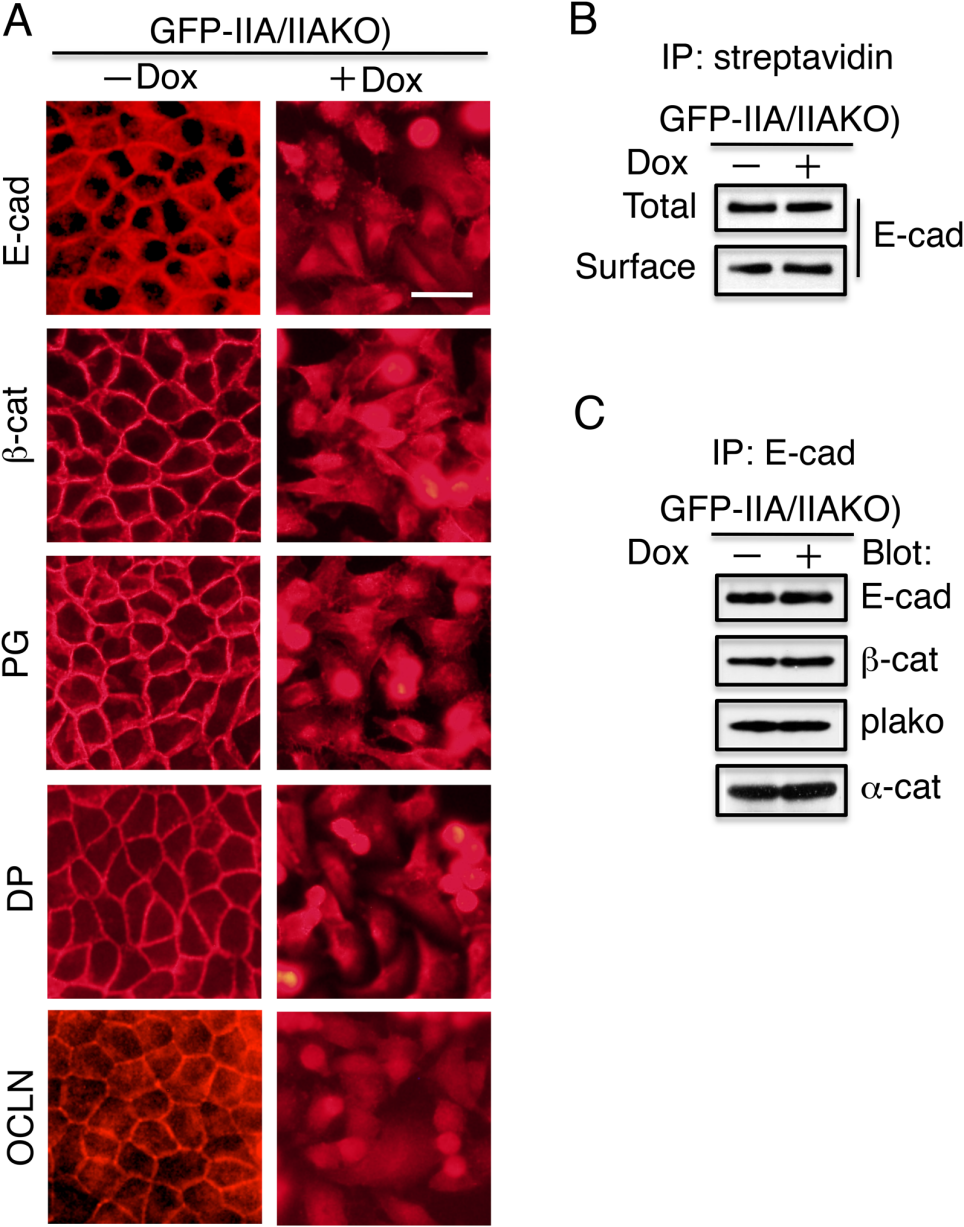
**GFP–IIA-dependent localization of the junction components to the cell–cell contact sites in IIAKO cells.** (A) Immunofluorescence staining of junction components. GFP–IIA/IIAKO cells were cultured for 4 days with (+) or without (−) Dox, and stained with antibodies against the following junction proteins: E-cadherin (E-cad), β-catenin (β-cat), plakoglobin (PG), desmoplakin (DP), or occludin (OCLN). In the absence of Dox [(−) Dox], these components were detected at the cell–cell contact sites. In the presence of Dox, (+) Dox, none of these components was detected at contact sites. Scale bar: 25 µm. (B) Cell surface expression of E-cadherin does not require GFP–IIA. Cell surface proteins were biotinylated, and the biotinylated proteins were immunoprecipitated (IP) with streptavidin–agarose (avidin). The collected materials were separated on a gel, transferred to a membrane, and subsequently probed with anti-E-cadherin antibodies. Total cell lysates were used as loading controls. (C) NMIIA does not affect complex formation of E-cadherin with catenins. After lysis, E-cadherin was collected with anti-E-cadherin antibodies. The collected materials were separated on a gel, transferred to a membrane, and subsequently probed with the indicated antibodies.

In the absence of Dox, when GFP–IIA was expressed, the components of cell–cell junctions (E-cadherin, β-catenin, plakoglobin, desmoplakin, and occludin) were distributed predominantly at plasma membrane sites involved in lateral cell–cell contacts (Fig. 2A, left panels). Addition of Dox not only reduced GFP–IIA expression and induced morphological changes, but also caused these components to move from cell–cell contact sites on the plasma membrane to an even distribution throughout the cell (Fig. 2A, right panels).

To monitor the presence of E-cadherin on the outer side of the plasma membrane, we selectively biotinylated the cell surface of GFP–IIA/IIAKO cells cultured in the absence or presence of Dox with a membrane-impermeable reagent, sulfoNHS-biotin. Biotinylated proteins were recovered with immobilized avidin and subjected to immunoblotting with antibodies against E-cadherin (Fig. 2B). E-cadherin was expressed at the cell surface irrespective of the addition of Dox (Fig. 2B). These data demonstrate that the established cell line failed to assemble junction components due to the absence of GFP–IIA.

Complex formation of E-cadherin with catenins, β-catenin, plakoglobin, and α-catenin is required for the assembly of junction complexes. Therefore, we investigated the association of E-cadherin with catenins. E-cadherin was collected by immunoprecipitation using E-cadherin antibodies, and after separation and transfer to nitrocellulose membrane the co-precipitated materials were probed with antibodies against catenins. E-cadherin isolated from cells cultured in the absence and presence of Dox collected similar amounts of β-catenin, plakoglobin, and α-catenin (Fig. 2C). Thus, the presence or absence of GFP–IIA had no effect on the association of E-cadherin with catenins.

### Correct localization of junction components requires α-catenin

**Fig. 3.**
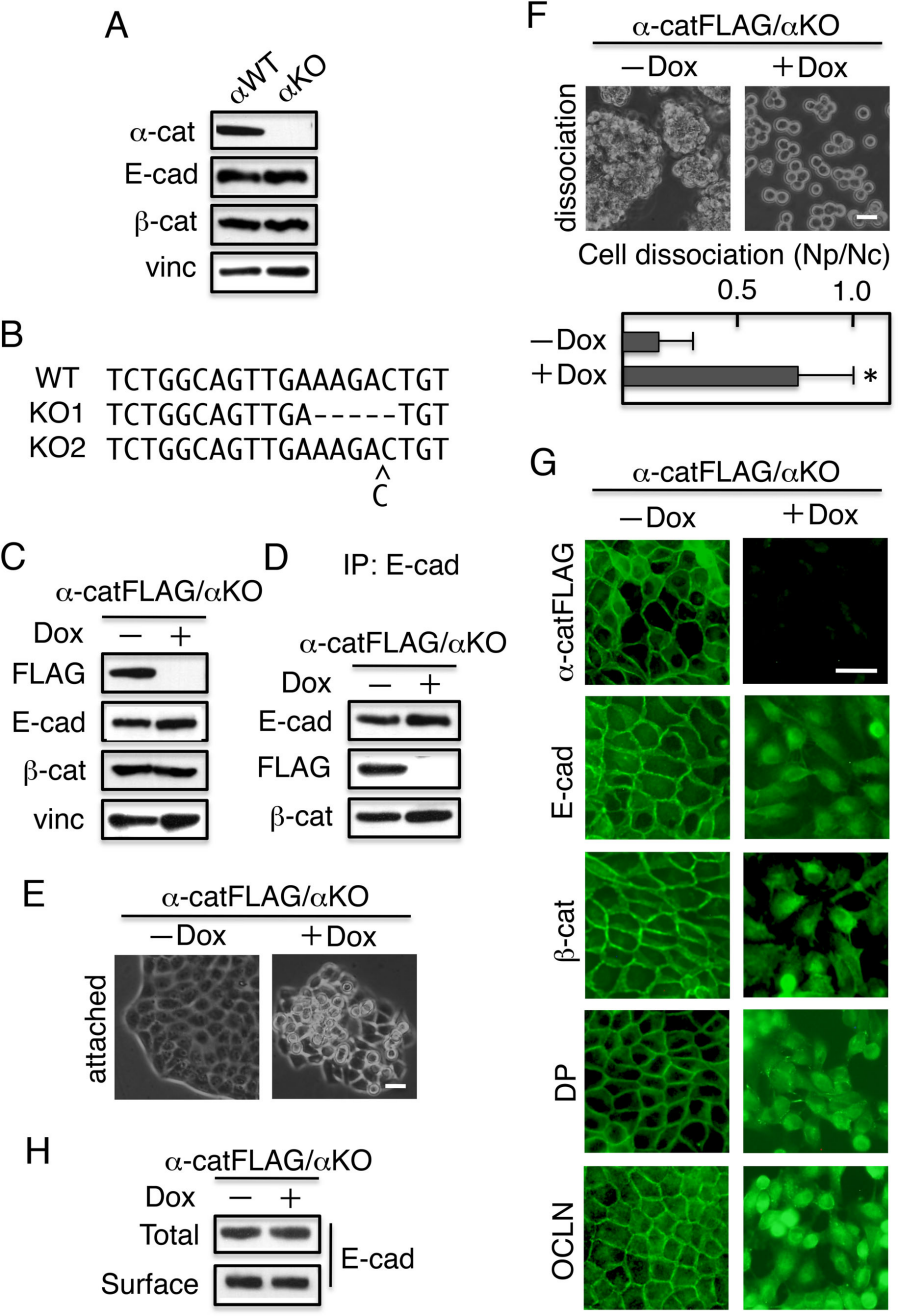
**Mislocalization of junction components in the absence of α-catenin.** Using the CRISPR/Cas9 system, the α-catenin gene of MDCK cells was ablated, yielding αKO cells, which were transfected with a vector encoding FLAG-tagged α-catenin (α-catFLAG) under the control of the TRE, yielding α-catFLAG/αKO cells. (A) Immunoblot detection of α-catenin (α-cat), E-cadherin (E-cad), β-catenin (β-cat), and vinculin (vinc) in αWT and αKO cells. Vinculin was used as a loading control. (B) Genomic sequencing revealed mutations in the target regions of α-catenin gene that induced frame-shift mutations. Of three independent clones sequenced, a 5-bp deletion was detected in two clones and a 1-bp insertion was identified in one clone. (C) Dox-repressible expression of FLAG-tagged α-catenin in α-catFLAG/αKO cells. The cells were cultured for 4 days with (+) or without (−) Dox, and subjected to immunoblot analysis with the indicated antibodies. α-catFLAG was detected with an anti-FLAG antibody. The addition of Dox repressed α-catFLAG expression, but did not affect the expression of other proteins. Vinculin was used as a loading control. (D) α-catFLAG co-immunoprecipitated with E-cadherin, along with β-catenin. (E) Cell morphology. Cells on the dish were observed using a phase-contrast microscope. (F) Dissociation assays. (Upper panels) Phase-contrast micrographs. Cells treated with Dox lost the mechanical integrity of their cell sheets, whereas untreated cells did not. (Bottom panels) Quantification of cell dissociation assays. Values represent the mean±s.e.; *n*=at least 3 times. *P<0.01 compared with control (−Dox). (G) Immunostaining. Cells were cultured and stained with antibodies against FLAG (α-catFLAG), E-cadherin (E-cad), β-catenin (β-cat), desmoplakin (DP), or occludin (OCLN). The addition of Dox disrupted localization of junction components at cell–cell contact sites. Scale bars: 25 µm. (H) Cell surface proteins were biotinylated, and the biotinylated proteins were immunoprecipitated (IP) with streptavidin–agarose (avidin). The collected materials were separated on a gel, transferred to a membrane, and subsequently probed with anti–E-cadherin antibodies. Total cell lysates were used as loading controls.

The failure of junction complex formation in IIAKO cells is also observed in cells lacking α-catenin expression, such as human colon cancer cells DLD-1/Δα (Taniguchi et al., 2005), raising the possibility that these molecules functionally interact. Therefore, we first established α-catenin KO MDCK cells using the CRISPR/Cas9 system; the resultant cells were termed αKO cells (Fig. 3A). Sequencing of genomic DNA isolated from the clone revealed either a 5-bp deletion or 1-bp insertion, resulting in a frame-shift mutation in the α-catenin gene (Fig. 3B).

The MDCK clone was then transduced with FLAG-tagged α-catenin construct under the control of TRE, yielding α-catFLAG/αKO cells. The cells were cultured for 4 days without or with Dox (−/+ Dox), and then analyzed by western blotting (Fig. 3C). FLAG-tagged α-catenin was detected in protein extracts from cells cultured without Dox, but not with Dox. When the same extracts were analyzed for expression of E-cadherin, β-catenin, and vinculin, we observed no significant difference in the amounts of these junction complex components, irrespective of the presence or absence of Dox (Fig. 3C). FLAG-tagged α-catenin was observed in complexes of E-cadherin containing β-catenin (Fig. 3D). In the absence of Dox, the cells exhibited epithelial morphology, but this morphology was disrupted by the presence of Dox (Fig. 3E). Cell–cell adhesion of α-catFLAG/αKO cells, assessed by the dissociation assays, was restored in the absence, but not the presence, of Dox (Fig. 3F). Thus the expression of FLAG-tagged α-catenin is required for cell–cell adhesion activity.

In the absence of Dox, the components of cell–cell junctions (E-cadherin, β-catenin, desmoplakin, and occludin) were distributed predominantly at plasma membrane sites involved in lateral cell–cell contacts (Fig. 3G). Addition of Dox not only decreased α-catenin expression and cell–cell adhesion, but also induced these components to move from cell–cell contact sites of the plasma membrane to an even distribution throughout the cell (Fig. 3G). Surface biotinylation analysis, however, revealed that E-cadherin was exposed on the plasma membrane of α-catFLAG/αKO cells, irrespective of the presence of Dox (Fig. 3H). These data demonstrate that the established cell line lost cell–cell adhesion activity due to ablation of α-catenin.

### α-catenin acts downstream of IIA

**Fig. 4.**
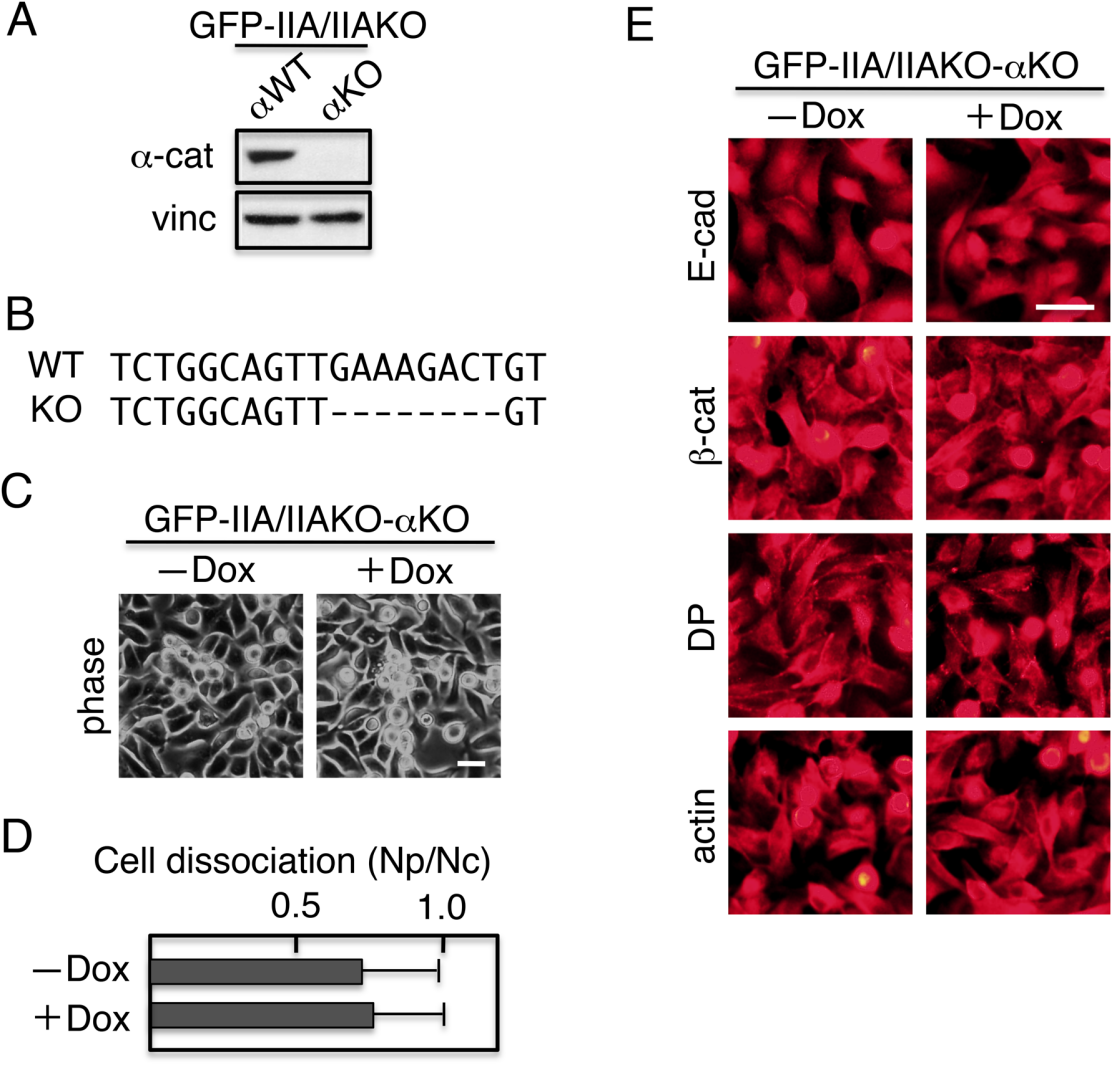
**KO of α-catenin gene in GFP–IIA/IIAKO cells results in mislocalization of adhesion junction components and inactivation of cell–cell adhesion irrespective of GFP–IIA expression.** Using the CRISPR/Cas9 system, the α-catenin gene of GFP–IIA/IIAKO cells was ablated, yielding GFP–IIA/IIAKO-αKO double-knockout cells. (A) Immunoblot detection of α-catenin (α-cat) and vinculin (vinc). GFP–IIA/IIAKO cells with wild-type α-catenin (αWT) were used as a positive control, and vinculin was used as a loading control. (B) Genomic sequencing revealed a deletion in the target region of the α-catenin gene that induced a frame-shift mutation. An 8-bp deletion was identified in all six clones sequenced. (C) Morphology of GFP–IIA/IIAKO-αKO cells. (D) Dissociation assays. Cells cultured in the presence or absence of Dox were subjected to dissociation assays, and dissociation was quantified. Values represent the mean±s.e.; *n*=at least 3 times. (E) Distribution of adhesion components. Cells were stained with antibodies against E-cadherin (E-cad), β-catenin (β-cat), desmoplakin (DP), or TRITC-labeled phalloidin (actin). Scale bars: 25 µm.

IIAKO and αKO cells exhibit highly similar properties in regard to assembly of the adhesion complex. To elucidate the molecular interaction of IIA and α-catenin, we ablated the α-catenin gene in GFP–IIA/IIAKO cells. The resultant cells, termed GFP–IIA/IIAKO-αKO, did not express α-catenin (Fig. 4A). Sequencing of genomic DNA isolated from the clone revealed an 8-bp deletion in the α-catenin gene, which results in a frame-shift mutation (Fig. 4B). When these cells were cultured in the absence or presence of Dox, the cell morphology was not observed irrespective of the presence of Dox (Fig. 4C). The integrity of cell junctions was also lost, as determined by dissociation assays (Fig. 4D). Moreover, the components of the adhesion complex were not assembled at cell–cell contact sites of the cell surface (Fig. 4E). These results show that α-catenin acts downstream of IIA in the assembly of junction components.

### Expression of α-catenin deletion constructs in IIAKO cells induces junction assembly and restores cell–cell adhesion

**Fig. 5.**
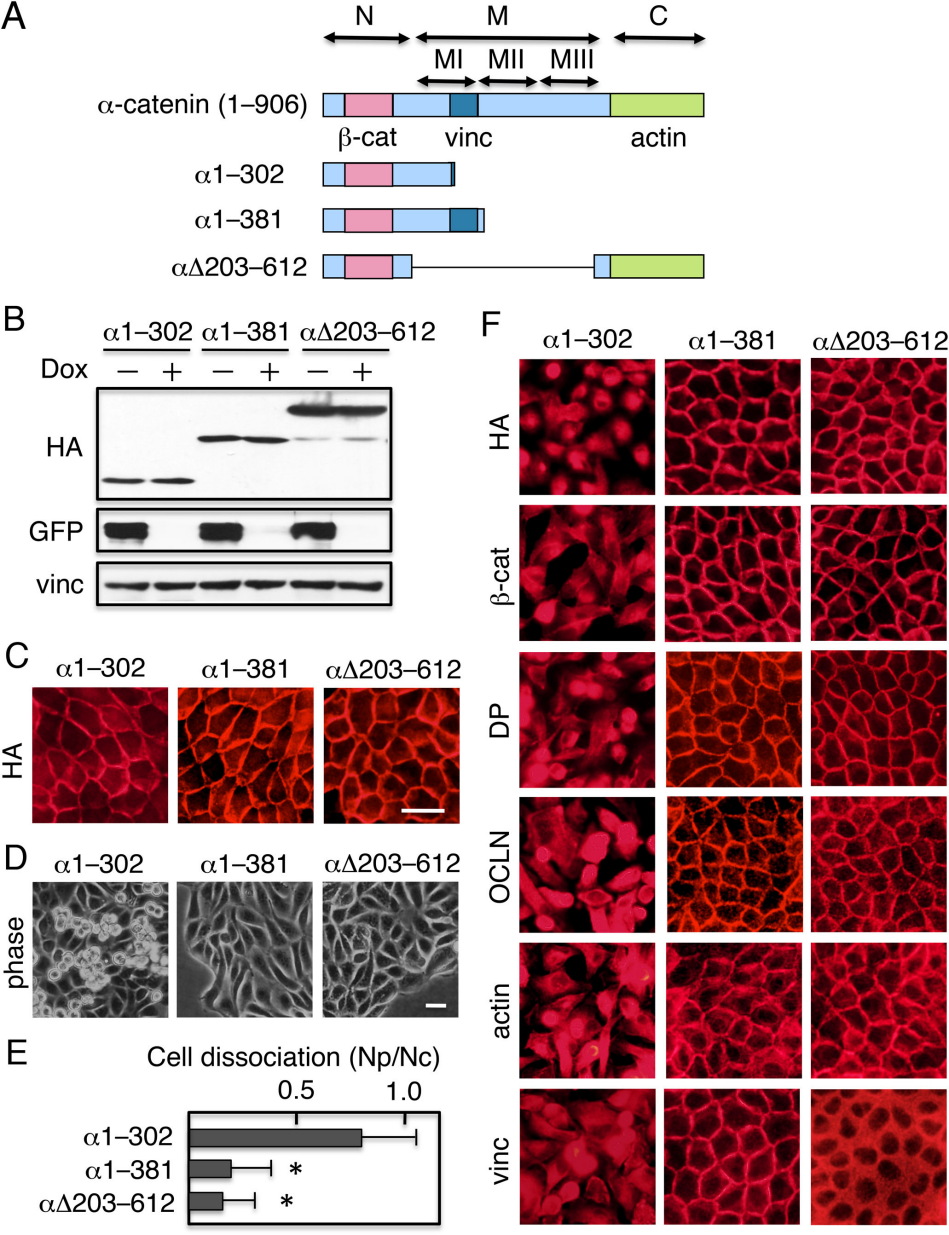
**Expression of α-catenin deletion constructs in IIAKO cells induces junction assembly and restores cell–cell adhesion.** (A) Schematic representation of α-catenin (top) and mutant derivatives (bottom). The N-terminal (N) domain binds to β-catenin. The middle (M) domain comprises three regions, MI, MII, and MIII. MI contains binding sites for vinculin, and MII and MIII are involved in regulation of force-dependent vinculin binding. The C-terminal (C) domain binds actin. α1–302 does not contain the vinculin- and actin-binding sites. α1–381 is a construct in which the carboxy-terminal 525 residues have been truncated, thereby removing the actin-binding site but retaining the vinculin-binding site. αΔ203–612 has an internal deletion of residues 203–612, and therefore lacks the vinculin-binding site but retains the actin-binding site. All constructs were tagged with HA. (B) Immunoblot detection of α-catenin polypeptides. GFP–IIA/IIAKO cells expressing α1–302, α1–381, or αΔ203–612 were cultured in the presence or absence of Dox for 4 days, and then analyzed. The blots were probed with anti-HA, anti-GFP, and anti-vinculin antibodies. (C) Immunofluorescence staining with anti-HA revealed that these constructs were localized at cell–cell contact sites of cells cultured in the absence of Dox. (D) Morphology of GFP–IIA/IIAKO cells expressing α1–302, α1–381, or αΔ203–612. Cells cultured in the presence of Dox to suppress GFP–IIA expression, and were observed using a phase contrast microscope. (E) Dissociation assays. Cells were incubated with Dispase, and then detached cells were subjected to mechanical stress by pipetting and quantified. Values represent the mean±s.e.; *n*=at least 3 times. *P<0.01 compared with control (cells expressing α1–302). (F) Distribution of the junction components. Dox-treated cells were stained with antibodies against HA, E-cadherin (E-cad), β-catenin (β-cat), desmoplakin (DP), occludin (OCLN), or vinculin (vinc). TRITC-phalloidin was used to detect actin. Scale bars: 25 µm.

α-catenin consists of three domains (Fig. 5A) (Ishiyama et al., 2013; Yao et al., 2014; Escobar et al., 2015; Kang et al., 2017). The N-terminal (N) domain binds to β-catenin. The middle (M) domain comprises three regions, MI, MII, and MIII. MI contains binding sites for vinculin, and MII and MIII are involved in regulation of force-dependent vinculin binding and are collectively termed the inhibitory region (Yonemura et al., 2010). The C-terminal (C) domain binds actin. We previously reported that expression of not only full-length α-catenin, but also α-catenin constructs with internal or C-terminal deletions, in α-catenin-deficient DLD-1 cells results in acquisition of cell–cell adhesion and formation of both adherens junction and desmosomes (Ozawa, 1998; Matsubara and Ozawa, 2001; Taniguchi et al., 2005). α1–381 is a construct in which the carboxy-terminal 525 residues are ablated by a truncation that removes the actin-binding site and inhibitory region but retains the vinculin-binding site (Fig. 5A). α1–302, which has an additional deletion in the vinculin-binding site, was used as a negative control. αΔ203–612 has an internal deletion of residues 203–612, and therefore lacks the entire M domain and thus the vinculin-binding site, but still retains the actin-binding site. We assumed that if NMIIA induces conformational changes in α-catenin and thereby activates it, these deletion constructs could act as activated α-catenin molecules in cells lacking IIA expression. GFP–IIA/IIAKO cells were transfected with expression vectors for these constructs; stable transfectants were isolated, cultured in the presence or absence of Dox for 4 days, and then analyzed. Immunoblot analysis revealed that although the level of GFP–IIA was decreased by addition of Dox, the levels of the deletion constructs were not (Fig. 5B). Cells expressing these constructs exhibited epithelial morphology in the absence of Dox (not shown), and the constructs were detected at cell–cell contact sites (Fig. 5C). Cells expressing α1–302 lost epithelial morphology in the presence of Dox, whereas cells expressing either α1–381 or αΔ203–612 retained this morphology (Fig. 5D). Dissociation assays of cells cultured in the presence of Dox revealed that cells expressing α1–302 were sensitive to mechanical dissociation, whereas cells expressing either α1–381 or αΔ203–612 were resistant (Fig. 5E). Consistent with these observations, α1–302 and the components of the junction complex were no longer detected at cell–cell contact sites in cells expressing α1–302 after culture with Dox, whereas in the cells expressing either α1–381 or αΔ203–612, these constructs and the junction components remained at cell–cell contact sites even after culture with Dox (Fig. 5F). Of note, the distribution of vinculin differed among cells expressing different constructs. Vinculin was detected at cell–cell contact sites in cells expressing α1–381, but in the cytoplasm in cells expressing αΔ203–612 (Fig. 5F, bottom panels). Thus α1-381 mutant protein restores α-catenin function by recruiting vinculin in the absence of NMIIA activity.

**Fig. 6.**
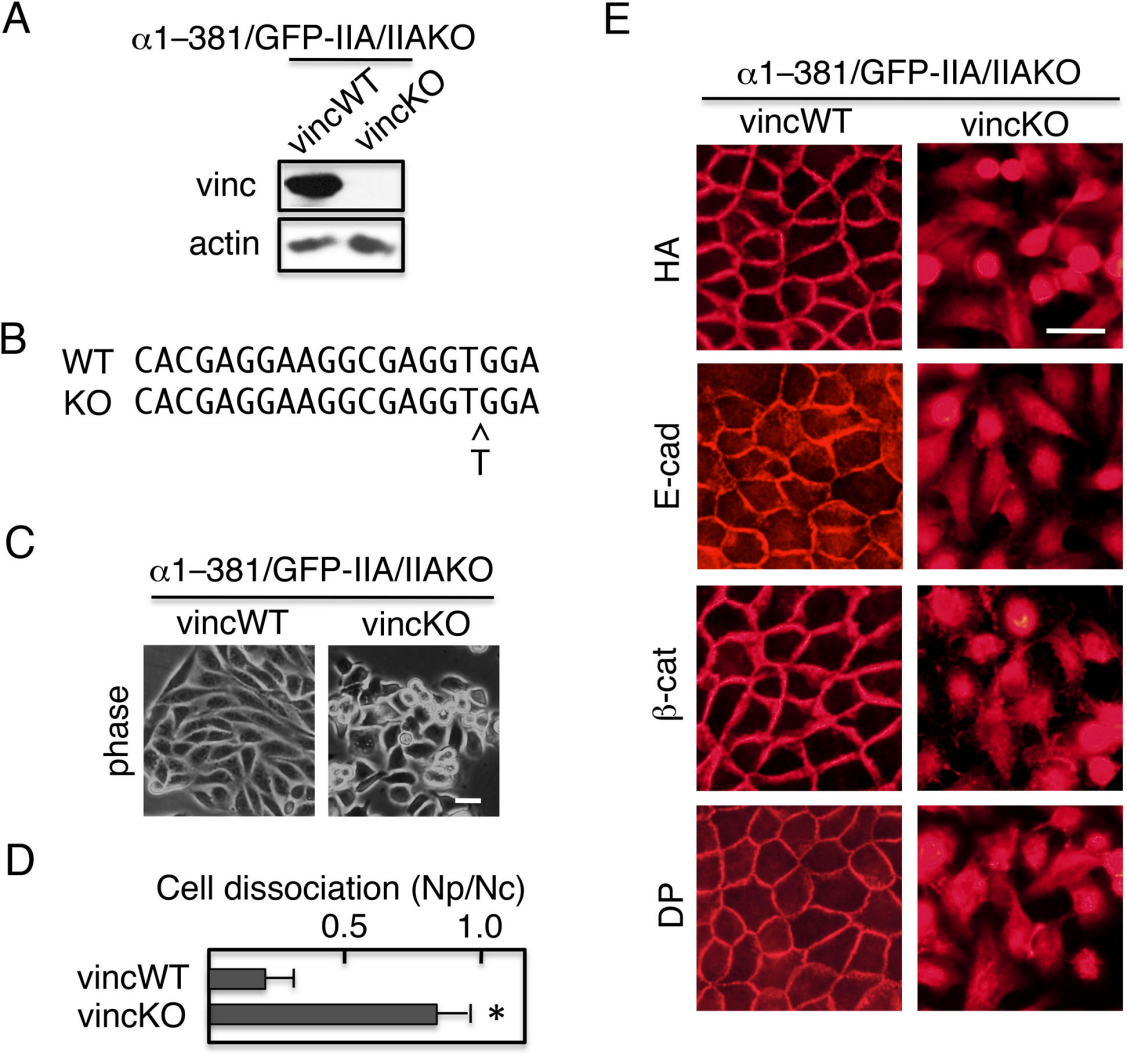
**The α-catenin construct α1–381 requires vinculin for its ability to rescue junction formation.** Using the CRISPR/Cas9 system, the vinculin gene was ablated in α1–381/GFP–IIA/IIAKO cells, yielding α1–381/GFP–IIA/IIAKO-vincKO double-knockout cells. (A) Immunoblot detection of vinculin (vinc) and actin. α1–381/GFP–IIA/IIAKO cells with wild-type vinculin (vincWT) were used as a positive control, and actin was used as a loading control. (B) Genomic sequencing revealed a mutation in the target region of vinculin gene that induced a frame-shift. All 13 independent clones sequenced harbored the same insertion of 1 bp. (C) Morphology of α1–381/GFP–IIA/IIAKO-vincKO cells. Cells cultured in the presence of Dox were observed using a phase-contrast microscope. (D) Cells cultured in the presence of Dox were incubated with Dispase, and then detached cells were subjected to dissociation assays and quantified. Values represent the mean±s.e.; *n*=at least 3 times. *P<0.01 compared with control (vincWT cells). (E) Distribution of adhesion components. Cells were cultured in the presence of Dox, and then stained with antibodies against HA, E-cadherin (E-cad), β-catenin (β-cat), or desmoplakin (DP). Scale bars: 25 µm.

The recruitment of vinculin to cell–cell contact sites in cells expressing α1–381 strongly suggests that the action of α1–381 requires vinculin. To investigate this possibility, the vinculin gene was ablated using the CRISPR/Cas9 system in GFP–IIA/IIAKO cells expressing α1–381. Immunoblot analysis revealed that the vinculin knockout (vincKO) cells did not express vinculin (Fig. 6A). Genomic sequencing revealed a mutation in the target region of vinculin gene that induced a frame-shift (Fig. 6B). When the cells were cultured in the presence of Dox to suppress the expression of GFP–IIA, control wild-type vinculin (vincWT) cells exhibited epithelial morphology, whereas vincKO cells lost tight cell–cell contacts, as revealed by morphological observation (Fig. 6C). Dissociation assays confirmed that vincKO cells lost tight cell–cell contact (Fig. 6D). Under these conditions, the components of cell junctions were not assembled at cell–cell contact sites (Fig. 6E, right panels). Thus, the presence of vinculin is required for the action of α1–381.

### Expression of an E-cadherin–α-catenin chimeric molecule restores cell–cell adhesion of cells lacking IIA expression

**Fig. 7.**
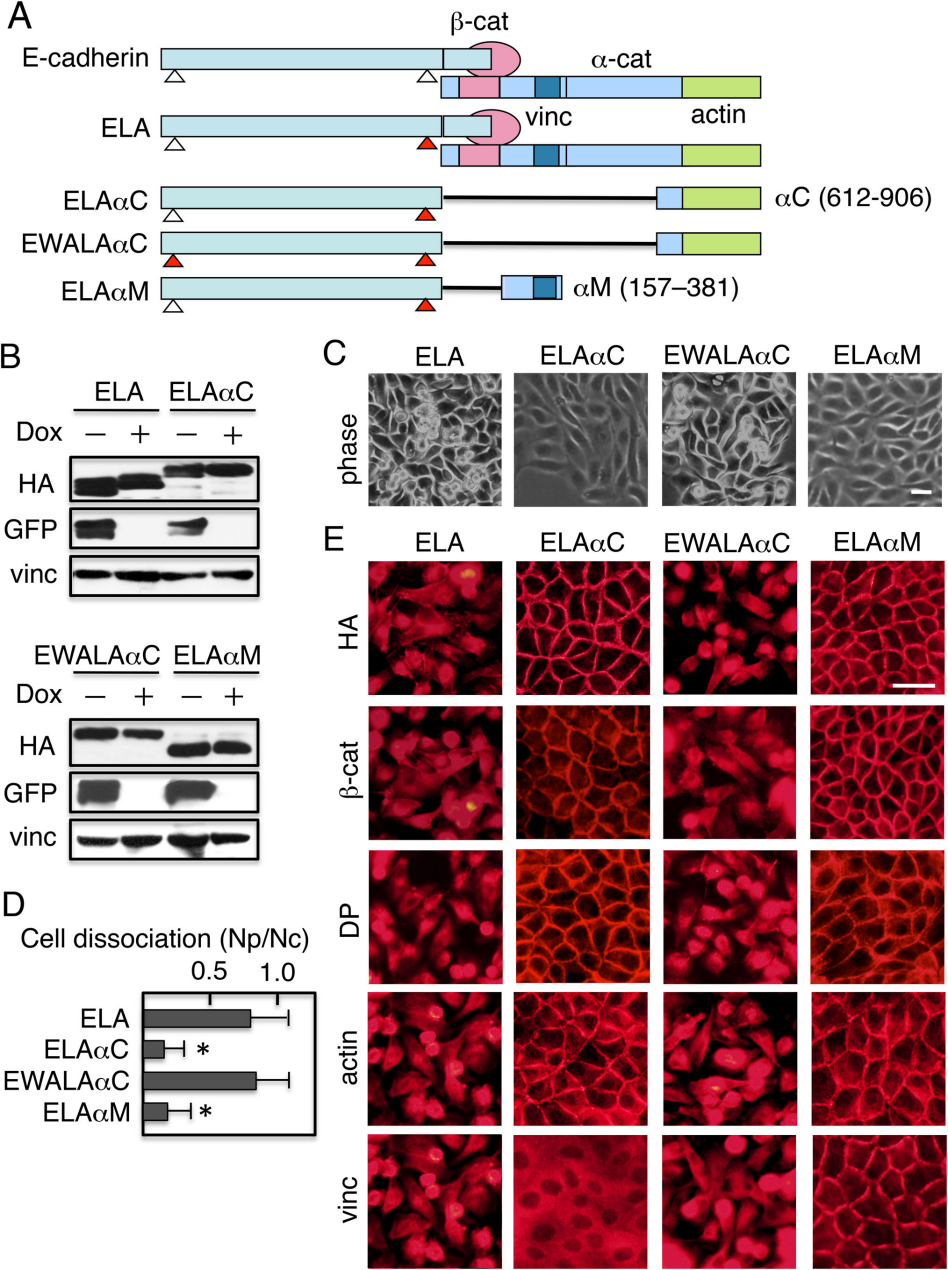
**E-cadherin–α-catenin chimeric molecules restore cell–cell adhesion and junction assembly.** (A) Schematic representation of E-cadherin and its four derivatives. E-cadherin associates with catenins (α-cat and β-cat). ELA is a mutant E-cadherin in which two leucine residues at positions 587 and 588 were replaced with alanine residues. This substitution improves the cell-surface localization of E-cadherin. ELAαC is an ELA–α-catenin chimeric protein consisting of (a) the entire extracellular and transmembrane domains of E-cadherin, as well as the first 80 amino acids of its cytoplasmic domain, excluding the region required for β-catenin–binding, and (b) α-catenin regions encompassing amino acids 612–906 (see Fig. 5A). EWALAαC has an additional mutation of the Trp2 to alanine (W2A) in the E-cadherin extracellular domain, which abolishes lateral dimerization and the adhesive interactions of E-cadherin. ELAαM is another ELA–α-catenin chimeric protein containing α-catenin regions encompassing amino acids 157–381, which include the region necessary for association with vinculin. All constructs were tagged with HA. (B) Immunoblot detection of ELA, ELAαC, EWALAαC, and ELAαM chimeras expressed in GFP–IIA/IIAKO cells. Cells expressing the respective chimeras were cultured in the presence or absence of Dox for 4 days, and cell lysates prepared were analyzed. Blots were stained with anti-HA, anti-GFP, or anti-vinculin antibodies. Vinculin was used as a loading control. (C) Morphology of cells expressing the chimeras. Cells were cultured in the presence of Dox to suppress GFP–IIA expression, and then observed using a phase-contrast microscope. (D) Cells cultured in the presence of Dox were incubated with Dispase, and then detached cells were subjected to dissociation assays and quantified. Values represent the mean±s.e.; *n*=at least 3 times. *P<0.01 compared with control (cells expressing ELA). (E) Distribution of adhesion components. Cells cultured in the presence of Dox were stained with anti-HA, anti–β-catenin (β-cat), anti-desmoplakin (DP), TRITC-labeled phalloidin (actin), or anti-vinculin (vinc). Scale bars: 25 µm.

Chimeric proteins composed of E-cadherin and α-catenin function as cell adhesion molecules and can rescue the loss of E-cadherin, β-catenin, or α-catenin (Nagafuchi et al., 1994; Ozawa, 1998; Ozawa and Kobayashi, 2014). To obtain further insight into the role of IIA in cadherin-mediated cell adhesion, we expressed mutant E-cadherin (ELA) or an E-cadherin–α-catenin chimera (ELAαC, EWALAαC, or ELAαM) in GFP–IIA/IIAKO cells (Fig. 7A). ELA is a mutant E-cadherin in which the two leucine residues at positions 587 and 588 were replaced with two alanine residues (LA substitution). This substitution increases the cell-surface localization of E-cadherin when β-catenin is not available (Ozawa and Kobayashi, 2014). ELAαC, a chimera composed of C-terminally truncated E-cadherin and the C-terminal one-third of the α-catenin polypeptide (residues 612–906), does not interact with β-catenin, but still functions as a cell adhesion molecule (Ozawa and Kobayashi, 2014). The second chimera (EWALAαC) has an additional mutation of Trp2 to alanine (W2A) in the E-cadherin extracellular domain, which abolishes lateral dimerization and the adhesive interactions of E-cadherin (Ozawa, 2002). ELAαM is another ELA–α-catenin chimeric protein with α-catenin regions encompassing amino acids 157–381, which contain the domains necessary for association with vinculin. Like ELAαC, ELAαM functions as a cell adhesion molecule (Ozawa and Kobayashi, 2014). GFP–IIA/IIAKO cells were transfected with expression vectors for these constructs; stable transfectants were isolated, cultured in the presence or absence of Dox for 4 days, and then subjected to analysis. Immunoblot analysis revealed that although the level of GFP–IIA was decreased by addition of Dox, the levels of the constructs were not (Fig. 7B). Expression of these constructs in the cells cultured in the absence of Dox, when GFP–IIA was expressed, did not induce any morphological changes (data not shown). Although expression of ELA in GFP–IIA− cells cultured in the presence of Dox did not rescue cells undergoing morphological changes, expression of ELAαC, but not that of EWALAαC, a mutant chimera without adhesion activity, prevented the morphological changes induced by the addition of Dox (Fig. 7C). The third chimera (ELAαM) also prevented the morphological changes induced by the addition of Dox (Fig. 7C). We then analyzed the integrity of cell junctions in dissociation assays. GFP–IIA/IIAKO cells expressing ELAαC or ELAαM exhibited a significant degree of integrity, whereas expression of ELA or EWALAαC had no effect (Fig. 7D). Thus, ELAαC or ELAαM expression in GFP–IIA− cells restored assembly of cell junctions in the absence of NMIIA.

When GFP–IIA/IIAKO cells expressing ELAαC or ELAαM were stained with antibodies against HA, β-catenin, or desmoplakin, these proteins were detected at the cell–cell contact sites of the membrane, whereas in GFP–IIA/IIAKO cells expressing ELA or EWALAαC, these proteins were not localized at these sites (Fig. 7E). Actin stained with TRITC-labeled phalloidin was detected at cell–cell contact sites of cells expressing ELAαC or ELAαM, but not those of cells expressing ELA or EWALAαC. Consistent with the results obtained with the α-catenin deletion constructs, vinculin was detected at cell–cell contact sites in cells expressing ELAαM, but not those expressing other constructs (Fig. 7E, bottom panels). Thus the ELAαM chimera, which contains only the vinculin-binding site of α-catenin, recruited vinculin to cell–cell contact sites and rescued the defects resulting from NMIIA deficiency.

### GFP–IIA/IIAKO cells lacking the vinculin gene retain the ability to assemble junction complexes

**Fig. 8.**
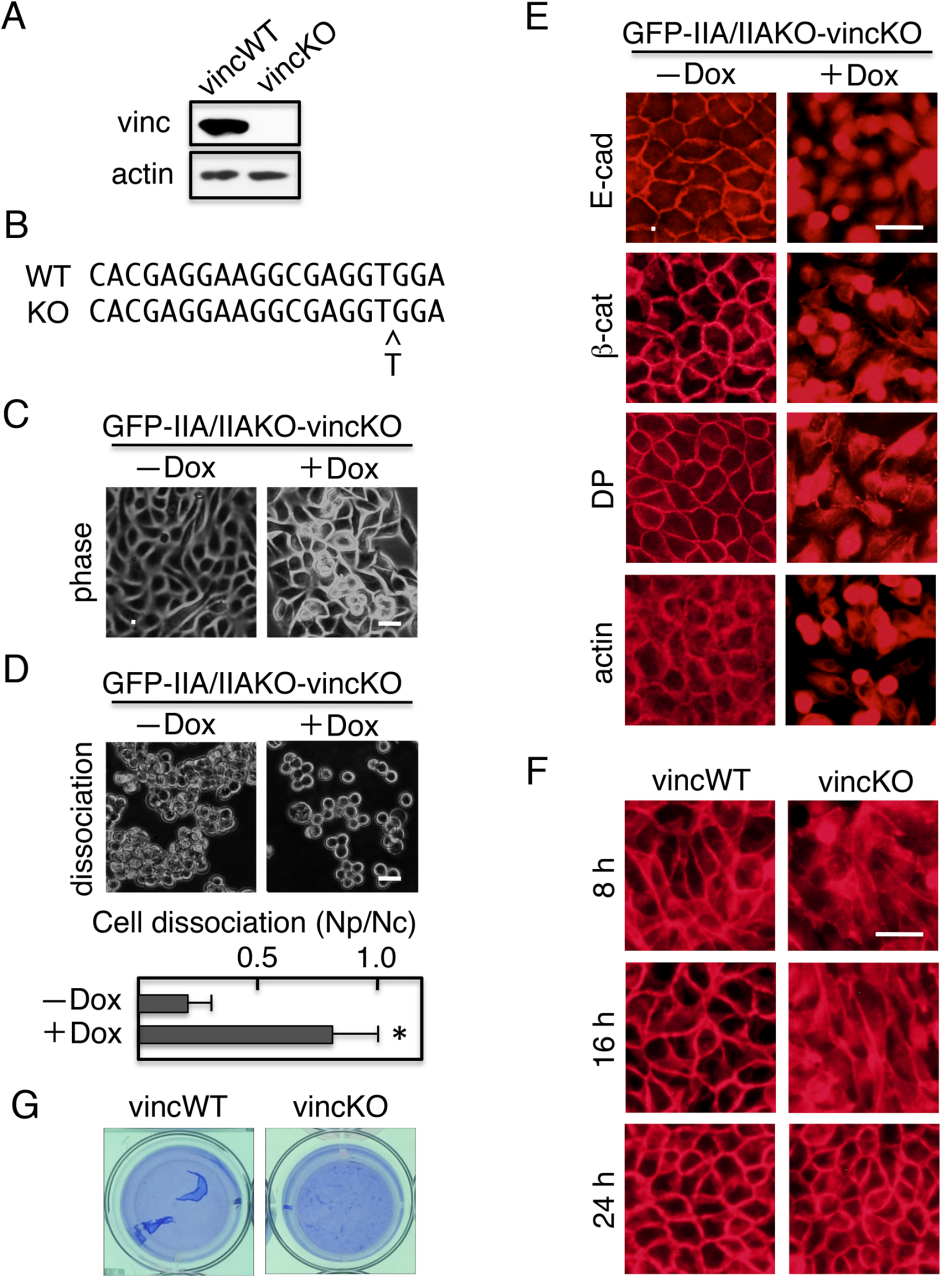
**GFP–IIA/IIAKO cells lacking the vinculin gene retain the ability to assemble junction complexes.** Using the CRISPR/Cas9 system, the vinculin gene was ablated in GFP–IIA/IIAKO cells, yielding GFP–IIA/IIAKO-vincKO double-knockout cells. (A) Immunoblot detection of vinculin (vinc), and actin. GFP–IIA/IIAKO cells with wild-type vinculin (vincWT) were used as a positive control, and actin was used as a loading control. (B) Genomic sequencing revealed mutation in the target region of vinculin gene that induces frame-shift. All 13 independent clones sequenced harbored the same 1-bp insertion. (C) Morphology of GFP–IIA/IIAKO-vincKO cells. Cells cultured in the presence or absence of Dox were observed using a phase-contrast microscope. (D) Dissociation of GFP–IIA/IIAKO-vincKO cells. Cells cultured in the presence or absence of Dox were subjected to dissociation assays. (Upper panels) Phase contrast micrographs. Cells treated with Dox lost the mechanical integrity of their cell sheets, whereas untreated cells did not. (Bottom panels) Quantification of cell dissociation assays. Values represent the mean±s.e.; *n*=at least 3 times. *P<0.01 compared with control (−Dox). (E) Distribution of adhesion components. Cells were stained with antibodies against E-cadherin (E-cad), β-catenin (β-cat), desmoplakin (DP), or TRITC-labeled phalloidin (actin). (F) Kinetics of junction formation. VincWT cells and vincKO cells were plated on coverslips and allowed to form junctions. After incubation for the indicated times, cells were fixed and stained with anti–β-catenin antibodies. (G) Cell sheets formed by vincKO cells dissociated easily. Cells were cultured for 24 h without Dox, incubated with Dispase, and then fixed as but without mechanical stress by pipetting. Scale bars: 25 µm.

Although an α-catenin construct containing only the vinculin-binding site could establish the junction complexes in cells without IIA expression, an α-catenin construct with only the actin-binding site also had this ability, raising the possibility that vinculin is not an absolute requirement for junction formation. To investigate this possibility, we ablated the vinculin gene in GFP–IIA/IIAKO using the CRISPR/Cas9 system, yielding GFP–IIA/IIAKO-vincKO double-knockout cells. Immunoblot analysis revealed that the cells did not express vinculin (Fig. 8A). Genomic DNA sequencing revealed a mutation in the target region of the vinculin gene that induced a frame-shift (Fig. 8B). When the cells were cultured in the absence of Dox, they exhibited epithelial morphology (Fig. 8C), and dissociation assays revealed that they could form cell sheets that were resistant to mechanical dissociation by pipetting (Fig. 8D). Immunostaining of cells revealed that the components of cell junctions were assembled at cell–cell contact sites (Fig. 7E, left panels). When expression of GFP–IIA was suppressed by culturing in the presence of Dox, the cells lost tight cell–cell contact, as revealed by morphological observation (Fig. 8C) and dissociation assays (Fig. 8D). Under these conditions, the components of cell junctions were not assembled at cell–cell contact sites (Fig. 8E, right panels). Although vincKO cells cultured for 2 days in the absence of Dox exhibited almost identical morphology (Fig. 8C) and distribution of junction components in comparison with vincWT cells (Fig. 8E), the kinetics of junction assembly were much slower in vincKO than in vincWT (Fig. 8F). Thus, the presence of vinculin accelerates junction formation. Cell sheets formed by vincKO cells dissociated more easily than those formed by vincWT cells, as confirmed by detachment of cell sheets with Dispase (Fig. 8G).

## DISCUSSION

In this study, we ablated the NMHCIIA (IIA) gene (*MYH9*) in MDCK cells, and then introduced GFP-tagged IIA (GFP–IIA) under the control of the Tet promoter. Thus, we could control the expression of GFP–IIA by culturing cells in the presence or absence of Dox. Specifically, the cells expressed GFP–IIA in the absence of Dox but not in the presence of Dox, and could assemble the epithelial junction complex only in the former condition. Therefore, we conclude that expression of GFP–IIA is required for assembly of the junction complex. When the α-catenin gene was ablated in the same cells, they became unable to form the junction irrespective of the expression of GFP–IIA, strongly suggesting that α-catenin acts downstream of NMIIA.

Vinculin has been implicated in the anchoring of cadherin–catenin complexes to actin (Miyake et al., 2006; Maddugoda et al., 2007). Vinculin is a component of integrin-associated complexes (Geiger et al., 1980) and plays a role in mechanotransduction at focal adhesions Riveline et al. (2001). Vinculin is also found at AJs (Geiger et al., 1985) and acts as a binding partner for α-catenin in mature cadherin-based AJs (Watabe-Uchida et al., 1998). Vinculin binds to α-catenin in response to conformational changes in both proteins; this, in turn, leads to the formation of a putative heterotetramer in which both the vinculin and α-catenin C-terminal domains bind F-actin with high affinity (Rangarajan and Izard, 2012; Choi et al., 2012). Structural and biochemical analyses have suggested that α-catenin undergoes a conformational change from the closed to open conformation, thereby unmasking the vinculin-binding domain (Yang et al., 2001; Pokutta et al., 2002). This conformational change of α-catenin, controlled by a force-dependent mechanism, may be responsible for myosin II-dependent recruitment of vinculin in mature AJs (Miyake et al., 2006; Maddugoda et al., 2007; le Duc et al., 2010; Yonemura et al., 2010). Indeed, recent experimental data suggest that cadherin is constitutively under actomyosin-generated tension (Smutny et al., 2010; Borghi et al., 2012). It is generally assumed that myosin II-generated tension induces a conformational change in α-catenin, uncovering a vinculin-binding site. Vinculin is then recruited to AJs and becomes associated with more actin filaments, thus reinforcing cell–cell adhesion (Yonemura et al., 2010; le Duc et al., 2010; Yao et al., 2014; Kim et al., 2015). According to this paradigm, wild-type (WT) α-catenin in cells lacking myosin-II activity would not be stretched to expose the vinculin-binding site, and therefore would not interact with vinculin. By contrast, α-catenin mutant proteins with truncations that remove the putative auto-inhibitory domain would be predicted to have exposed vinculin-binding sites, and would thus interact with vinculin in a constitutive manner (Jurado et al., 2016). Consistent with this idea, we found that NMIIA KO cells expressing endogenous WT α-catenin failed to assemble junction components, but expression of not only GFP-tagged NMIIA, but also α1–381 mutant protein (which contains the vinculin-binding site), induced assembly of the complex. The C-terminally truncated forms of α-catenin that bind constitutively to vinculin strongly stabilize AJ dynamics (Chen et al., 2015; Yonemura et al., 2010; Thomas et al., 2013). Consistent with these observations, we observed accumulation of vinculin at cell–cell contact sites of cells expressing α1–381. Thus, α1-381 mutant protein restores α-catenin function by recruiting vinculin in the absence of NMIIA activity. Therefore, NMIIA acts as an activator of α-catenin by exposing its vinculin-binding site. In support of these results, the ELAαM chimera, which contains only the vinculin-binding site of α-catenin, also recruited vinculin to cell–cell contact sites and rescued the defects resulting from NMIIA deficiency.

Another construct, αΔ203–612, contains the actin-binding site but lacks the vinculin-binding site. αΔ203–612 could also restore α-catenin function in the absence of NMIIA activity. Expression of this construct in α-catenin–deficient cells induces assembly of junctions (Taniguchi et al., 2005). α-catenin mutants lacking the vinculin binding site can form actin-associated junctions in α-catenin–deficient cells (Huveneers et al., 2012; Twiss et al., 2012; Desai et al., 2013; Chen et al., 2015), which are completely devoid of vinculin (Chen et al., 2015). Because WT α-catenin is not activated without NMIIA activity, the truncation introduced in αΔ203–612 activated its actin-binding activity. Recent studies revealed strong and stable bonds between the cadherin–catenin complex and an actin filament form under force (Buckley et al., 2014). Therefore, the region deleted in the construct, aa 203–612 may also be involved in modulation of the actin-binding site. Thus, NMIIA acts as an activator of α-catenin by exposing not only the vinculin-binding site, but also the actin-binding site.

Although the ELAαC chimera could rescue the defects caused by lack of NMIIA activity, the EWALAαC chimera could not. The W2A substitution abolishes lateral dimerization of the extracellular domain of E-cadherin (Ozawa, 2002), and the chimeras do not have the N-terminal dimerization domain of α-catenin; consequently, this chimera remains as a monomer. Because the monomeric E-cadherin–α-catenin chimera requires force-dependent binding to F-actin for induction of E-cadherin–mediated adhesion (Bianchini et al., 2015), EWALAαC requires NMIIA activity, and therefore cannot rescue the defect due to the absence of NMIIA. Because the W2A mutation also abolishes adhesive interactions, the absence of this activity could explain the failure to rescue.

Disruption of the gene encoding E-cadherin (Larue et al., 1994; Riethmacher et al., 1995) or α-catenin (Torres et al., 1997) inhibits formation of the trophectoderm, the first epithelial tissue to differentiate in the mouse embryo, thereby preventing preimplantation development. Vinculin-null embryos are also lethal, but they survive until around embryonic day 10 (Xu et al., 1998). Therefore, at the early stages of development, the function of vinculin is dispensable. Depletion of vinculin alters AJs in MDCK cells (Sumida et al., 2011). In endothelial cells, vinculin does not play a crucial role during the formation and maintenance of intercellular adhesion, but does contribute to junction remodeling (Huveneers et al., 2012). Although the α1–381 deletion construct required vinculin for its ability to assemble cell junctions, the Δ203–612 construct, which lacks the vinculin-binding site, could also rescue the defect induced by the lack of myosin IIA activity. Consistent with this observation, ablation of the vinculin gene did not inhibit formation of cell junctions as long as NMIIA is present. Thus, knockout of the α-catenin gene severely inhibited assembly of cell junctions, but knockout of vinculin in MDCK cells did not perturb the junction formation, indicating that the development of the cadherin-based adhesion complex requires α-catenin but not vinculin. However, we did observe a delay in junction formation in vincKO cells. Furthermore, the cell sheets formed by vincWT cells were much less susceptible to fragmentation than those of vincKO cells. Thus, vinculin is required for the stabilization of the cell sheets. We hypothesize that even during the formation of intercellular adhesion, assembly and disassembly of components take place, and vinculin shifts the equilibrium toward formation by stabilizing the junctions.

## MATERIALS AND METHODS

### CRISPR/Cas9 plasmid

For CRISPR/Cas9-mediated knockout of the genes of interest (Cong et al., 2013; Mali et al., 2013), we used the pCGsapI vector developed by Takayuki Sakurai (Shinshu University). The vector contains the *hCas9* gene under the control of the CAG promoter and a unique cloning site, *Sap*I site, for insertion of the guide RNA under the control of the U6 promoter. Therefore, all synthetic oligonucleotides corresponding to the guide RNA and complementary chain contain the adaptor sequence for *Sap*I. The following oligonucleotides were used to construct guide RNAs (lowercase letters represent the adaptor sequence: NMIIA, accgCCCACCCAAGTTCTCCAAGGg and aaacCCTTGGAGAACTTGGGTGGGc; α-catenin, accgTCTGGCAGTTGAAAGACTGTg and aaacACAGTCTTTCAACTGCCAGAc); vinculin (accgCACGAGGAAGGCGAGGTGGAg and aaacTCCACCTCGCCTTCCTCGTGc).

### Identification of mutations induced by the CRISPR/Cas9 system

Genomic DNA was isolated from each clone negative for α-catenin, NMIIA, or vinculin, as determined by immunoblot analysis. DNA fragments covering the gRNA target regions were amplified using the following combinations of primers: NMHCIIA, CCGATAAGTATCTCTATGTGGA/TTCCTTGAGGTTGTGCAACAC and AAACTTCATCAATAACCCGCTG/TAGATGAGCCCTGAGTAGTAG; α-catenin, AGTTACTGGGTTCTCTAGTGC/CTGCAGAGTCCTACCTGTGT and CCATCTAGAATGTAGCTGTGC/TGCTCTGTATTTGTTTCCTAGG; and vinculin, TGTTTCATACGCGCACGATC/AAGGTCACAGAGCAGAGGAG and ACGATCGAGAGCATCTTGGA/CTCGCTCAAGGTCACAGAG. The resultant PCR products were cloned into pGEM-T and transformed into *E. coli*. Plasmid DNA, isolated from multiple colonies arising from each transformation, was sequenced. Multiple clones of two different sequences were obtained for the NMIIA and α-catenin genes isolated from MDCK(IIAKO) and MDCK(αKO) cells, respectively. However, multiple clones of only one sequence were isolated for the α-catenin and vinculin genes isolated from GFP–IIA/IIAKO-αKO, α1–381/GFP–IIA/IIAKO-vincKO, and GFP–IIA/IIAKO-vincKO cells, respectively.

### Expression vector construction

Expression vectors containing the HA-tagged α-catenin mutant were previously described (Ozawa, 1998; Matsubara and Ozawa, 2001). A CAG vector containing HA-tagged α-catenin (pC-αHA) (Taniguchi et al., 2005) was digested with *Not*I and *Eco*RV, and the fragment containing full-length α-catenin cDNA was cloned into the *Not*I/*Eco*RV site of pU-DNCT (Ozawa and Kobayashi, 2014), a derivative of pUHD10-3 (Gossen and Bujard, 1992), yielding pTRE-α-cateninFLAG. pCAGGShyg, which confers hygromycin resistance, and the pCAG/bsr-7 vector, which confers blasticidin resistance gene, were described previously (Ozawa and Kobayashi, 2014; Ozawa, 2015). pZeoSV2(+), which contains the zeocin-resistance gene, and the gRNA expression vector containing the α-1,3-galactosyltransferase gene were provided by Masahiro Sato (Kagoshima University). pTRE-GFP–NMHCIIA (Addgene plasmid # 10844) were gifts from Robert Adelstein (Wei and Adelstein, 2000).

### Cells and transfection

The Type II Madin-Darby canine kidney (MDCK) cell clone T23 (Barth et al., 1997) expressing the Tet repressor (provided by W. James Nelson, Stanford University) was cultured as described (Ozawa and Kobayashi, 2014). Cells were transfected with expression or targeting vectors (15 μg) together with drug-resistance vectors (1.5 μg) using the calcium phosphate precipitation method as previously described (Ozawa and Kobayashi, 2014). When multiple transfections were necessary, we used the Amaxa Nucleofector system (Amaxa GmbH, Cologne, Germany) and selected transfectants with either hygromycin (300 μg/ml), blasticidin (8 μg/ml), or Zeocin (1 mg/ml). Stable transfectants were identified by fluorescence microscopy and immunoblotting, and isolated as previously described (Ozawa and Kobayashi, 2014). At least three independent clones were selected for each construct to ensure that any observed effects were not due to phenotypic variability introduced by clonal selection (Fig. S1). To repress expression of GFP–NMIIA and α-catenin, cells were cultured in the presence of Dox (20 ng/ml) for 4 days. To isolate α1–381/GFP–IIA/IIAKO-vincKO double-knockout cells, α1–381/GFP–IIA/IIAKO cells were transfected along with the gRNA expression vectors targeting the vinculin and α-1,3-galactosyltransferase genes, and then selected with IB4 conjugated to saporin toxin (IB4-SAP) (Sato et al., 2014). IB4, an isolectin isolated from *Griffonia simplicifolia*, recognizes α-galactose residue found on the surface of cells. Thus, IB4-SAP kills cells expressing the WT α-1,3-galactosyltransferase gene (Thall et al., 1995), including MDCK cells, but not cells in which this gene has been ablated. The absence of α-galactose residues on the cell surface has no effect on the establishment of cell junctions (not shown).

### Antibodies

The following monoclonal antibodies were used to detect E-cadherin: DECMA-1, raised against the extracellular domain of E-cadherin (provided by Rolf Kemler, Max-Planck Institute for Immunobiology), and C20820, a mAb detecting the cytoplasmic domain of E-cadherin (BD Biosciences). For immunoprecipitation, rabbit polyclonal antibodies raised against the extracellular domain of E-cadherin (provided by Rolf Kemler) were used. Rat anti-HA mAb (3F10) was purchased from Roche Molecular Biochemicals (Mannheim, Germany). Mouse anti-FLAG (DYKDDDDK) mAb was purchased from Wako Pure Chemical Industries (Osaka, Japan). Mouse mAbs against α-catenin, β-catenin, and plakoglobin were purchased from BD Biosciences, and mAbs against vinculin and actin were obtained from Sigma-Aldrich. Rabbit anti–non-muscle myosin heavy chain II-A, II-B, and II-C antibodies were obtained from BioLegend (San Diego, USA). All secondary antibodies were obtained from Jackson ImmunoResearch Laboratories (West Grove, USA).

### Fluorescence microscopy

Immunofluorescence labeling of cells was performed as described (Ozawa and Kobayashi, 2014). In brief, cells were fixed with 3% paraformaldehyde in PBS for 20 min at room temperature, permeabilized with 0.1% Triton X-100, and then incubated with primary and secondary antibodies. Immunostained cells were analyzed on an Olympus fluorescence microscope (Tokyo, Japan) equipped with a CD72 camera (Olympus) or a confocal laser scanning microscope (Carl Zeiss Japan, Tokyo, Japan).

### Dissociation assay

Cells were washed with PBS, and then incubated for 1 h in DMEM supplemented with 10% FCS containing 2.4 U/ml of Dispase (Gibco). Detached cells were subjected to mechanical stress by pipetting with a 1-ml pipette. The extent of cell dissociation was represented by the index Np/Nc, where Np and Nc are the total numbers of particles and cells per dish, respectively. To obtain Nc, the detached cells were incubated for 10 min in the presence of 5 mM EGTA, and then subjected to mechanical stress as described (Ozawa and Kobayashi, 2014). Statistical analysis was performed by Student's *t*-test. Differences were considered to be significant at *P*<0.05.

### Cell surface biotinylation

Cells (5×10^5^) cultured on Transwell filters were incubated at 4°C twice with 0.5 mg/ml sulfo-NHS-biotin (Pierce Chemical, Rockford, USA) in both the upper and lower chamber. The labeled cells were washed with 50 mM NH_4_Cl in PBS at 4°C, stripped with 20 mM Tris-HCl (pH 8.0) containing 1% SDS, boiled for 3 min, passed four to five times through a 25-G needle, and finally mixed with nine volumes of 2% Triton X-100. Biotinylated proteins were collected using streptavidin-conjugated beads (Sigma-Aldrich).

### Immunoprecipitation and immunoblotting

Immunoprecipitation and immunoblot analyses were carried out as described (Ozawa and Kobayashi, 2014). In brief, cells (2×10^6^) were lysed in lysis buffer (25 mM Tris-HCl [pH 7.4], 1% Triton X-100, 2 mM EDTA, 10 mM sodium pyrophosphate, 10 mM NaF, 1 mM Na_3_VO_4_, 1 mM PMSF, 10 μg/ml leupeptin, and 25 μg/ml aprotinin). E-cadherin and associated proteins were collected using rabbit anti–E-cadherin antibodies pre-adsorbed onto protein G–Sepharose.

## Supplementary Material

Supplementary information
