# Supplementary material for: Nonmuscle myosin IIA is involved in recruitment of apical junction components through activation of α-catenin
Source: Biol Open. 2018 Apr 13;7(5):bio031369. doi: 10.1242/bio.031369 (PMC5992523; doi:10.1242/bio.031369)
Supplement: Supplementary information [file biolopen-7-031369-s1.pdf]

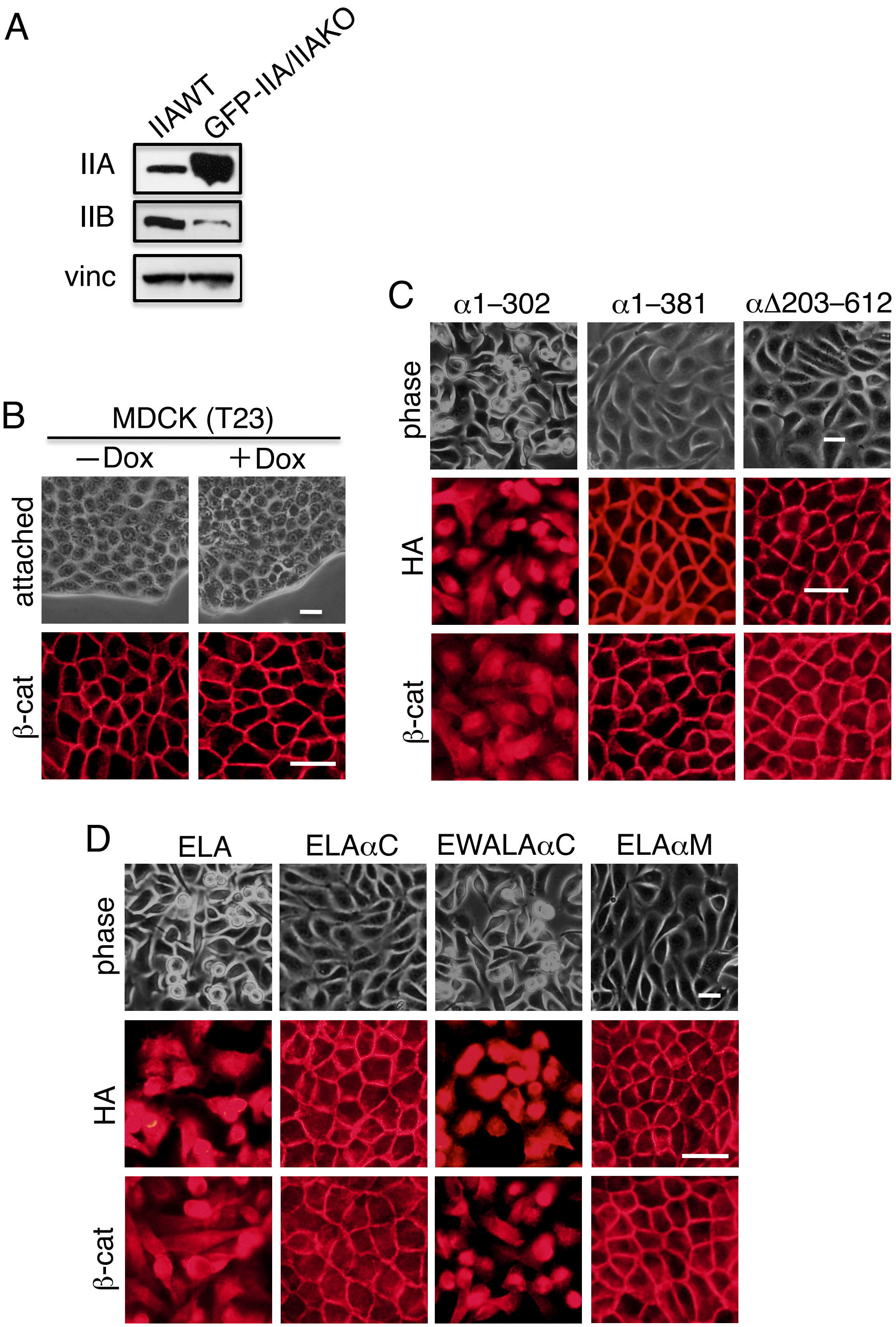

Biology Open • Supplementary information

Figure S1. (A) Downregulation of IIB expression as a result of GFP–IIA expression. Parental MDCK cells (IIAWT) and GFP–IIA/IIAKO cells were subjected to immunoblot analysis with the indicated antibodies. Endogenous IIA and GFP–IIA were detected with an anti-IIA antibody. The level of transduced GFP–IIA expression is very much high (2.5 times) as compared with that of endogenous NMIIA. The results were unexpected ones because GFP–IIA is under the control of tetracyclin-responsible element. Overexpression of GFP–IIA slightly downregulates NMIIB expression (~0.5). Vinculin was used as a loading control. (B) Doxycycline treatment has no effect on the morphology and the junction formation on control T23 MDCK cells. The cells were cultured for 4 days with (+) or without (−) Dox, and then were observed using a phase-contrast microscope (upper panels) or stained with anti-β-catenin antibody to examine the junction formation (lower panels). (C and D) To avoid phenotypic variability introduced by clonal selection, another cell clones expressing different constructs were examined for morphology and the junction formation. GFP–IIA/IIAKO cells expressing α-catenin deletion constructs, α1–302, α1–381, or αΔ203–612, were cultured in the presence of Dox to suppress GFP–IIA expression, and examined for morphology (C, upper panels) or stained with anti-HA or anti-β-catenin (β-cat). GFP–IIA/IIAKO cells expressing E-cadherin–α-catenin chimeras, ELA, ELAαC, EWALAαC, or ELAαM, were cultured in the presence of Dox, and were observed using a phase contrast microscope (D, upper panels) or stained with anti-HA or anti-β-catenin (β-cat). Bars, 25 µm.
